# Transgenic Clustered Regularly Interspaced Short Palindromic Repeat/Cas9-Mediated Viral Gene Targeting for Antiviral Therapy of Bombyx mori Nucleopolyhedrovirus

**DOI:** 10.1128/JVI.02465-16

**Published:** 2017-03-29

**Authors:** Shuqing Chen, Chengxiang Hou, Honglun Bi, Yueqiang Wang, Jun Xu, Muwang Li, Anthony A. James, Yongping Huang, Anjiang Tan

**Affiliations:** a Key Laboratory of Insect Developmental and Evolutionary Biology, Institute of Plant Physiology and Ecology, Shanghai Institutes for Biological Sciences, Chinese Academy of Sciences, Shanghai, China; b University of Chinese Academy of Sciences, Beijing, China; c Sericultural Research Institute, Jiangsu University of Science and Technology, Zhenjiang, Jiangsu, China; d Departments of Microbiology & Molecular Genetics and Molecular Biology & Biochemistry, University of California, Irvine, Irvine, California, USA; The Biodesign Institute, Arizona State University

**Keywords:** Bombyx mori nucleopolyhedrovirus, CRISPR/Cas9, transgenic silkworm, antiviral therapy

## Abstract

We developed a novel antiviral strategy by combining transposon-based transgenesis and the clustered regularly interspaced short palindromic repeats (CRISPR)/CRISPR-associated 9 (Cas9) system for the direct cleavage of Bombyx mori nucleopolyhedrovirus (BmNPV) genome DNA to promote virus clearance in silkworms. We demonstrate that transgenic silkworms constitutively expressing Cas9 and guide RNAs targeting the BmNPV *immediate early-1* (*ie-1*) and *me53* genes effectively induce target-specific cleavage and subsequent mutagenesis, especially large (∼7-kbp) segment deletions in BmNPV genomes, and thus exhibit robust suppression of BmNPV proliferation. Transgenic animals exhibited higher and inheritable resistance to BmNPV infection than wild-type animals. Our approach will not only contribute to modern sericulture but also shed light on future antiviral therapy.

**IMPORTANCE** Pathogen genome targeting has shown its potential in antiviral research. However, transgenic CRISPR/Cas9 system-mediated viral genome targeting has not been reported as an antiviral strategy in a natural animal host of a virus. Our data provide an effective approach against BmNPV infection in a real-world biological system and demonstrate the potential of transgenic CRISPR/Cas9 systems in antiviral research in other species.

## INTRODUCTION

The silkworm Bombyx mori, a completely domesticated and economically important insect, is susceptible to a variety of diseases. In China, almost 80% of silk cocoon crop losses are attributed to viral diseases (1). Bombyx mori nucleopolyhedrovirus (BmNPV) is a major viral pathogen that causes substantial economic losses to mass silkworm rearing in silkworm-raising countries (1, 2), and it still remains a big challenge to the sericulture industry without effective prevention methods. BmNPV is a member of the Baculoviridae family, which is characterized by a rod-shaped, enveloped virion containing a closed circular double-stranded DNA genome ∼130 kbp (kb) in length. Moving from a time- and labor-consuming traditional breeding approach, numerous transgenic (TG) strategies have been developed in modern sericulture to improve BmNPV resistance, although their practical use remains to be established. These TG methods are divided into two main types: overexpression of endogenous or exogenous antiviral genes (3, 4) and inhibition of viral genes through RNA interference (RNAi) (5, 6). Limited success has been achieved with the overexpression strategy. Only two antiviral genes, the endogenous antiviral gene Bmlipase-*1* and the exogenous antiviral gene *hycu-ep32*, have been reported to enhance the resistance of silkworms to BmNPV infection (3, 4). In contrast, TG RNAi has been applied extensively by silencing one or multiple essential BmNPV genes to destroy specific viral mRNAs (7, 8). It is important to select appropriate genes as targets in RNAi-based anti-BmNPV experiments, as silencing of different BmNPV genes results in variable antiviral resistance efficiency (9). Overall, moderate virus resistance has been reported for TG RNAi-based anti-BmNPV strategies, thus leaving the major challenge of how to inhibit viral DNA replication due to the persistence of BmNPV genomic DNA.

Recent advances in gene therapy strategies support pathogen genome targeting as a promising strategy in antiviral research. In contrast to the RNAi strategy, which works at a posttranscriptional level, pathogen genome targeting aims to directly disrupt pathology-causing viral DNA or RNA that persists or accumulates in host cells, thus eliminating viruses in the host. The clustered regularly interspaced short palindromic repeats (CRISPR)/CRISPR-associated 9 (Cas9) system, which has several advantages over homing endonucleases, zinc finger nucleases (ZFNs), and transcription activator-like effector nucleases (TALENs), has greatly facilitated the pathogen genome targeting strategy (10). The CRISPR/Cas9 system has been utilized to efficiently eradicate viruses in cell culture and mouse models of human disease, including the hepatitis B virus (HBV) (11, 12), Epstein-Barr virus (13, 14), and human immunodeficiency virus type 1 (HIV-1) in both preintegration and provirus stages (15, 16), providing initial evidence of the efficacy of CRISPR/Cas9 system-mediated antiviral therapy. The development of the RNA-targeting CRISPR/Cas9 system has expanded the scope of genetic engineering of pathogens to target RNA viruses, which have no DNA stages in their life cycles (17). Although it is a facile and efficient alternative to ZFNs and TALENs, the CRISPR/Cas9 system was reported to induce high-frequency off-target mutagenesis (18). Several approaches have been developed to improve the specificity of the guide RNA (gRNA)-Cas9 tool and accelerate its use for therapeutic applications (19–22), but more *in vivo* tests need to be conducted for a better understanding and nonbiased assessment of its applicability. Moreover, it is important and of general interest to demonstrate the efficacy of CRISPR/Cas9 system-mediated antiviral therapy in a legitimate, natural host-virus interaction.

We show here that a TG CRISPR/Cas9 system can be used as an anti-BmNPV strategy in a real-world biological system. We constructed a *piggy*Bac plasmid that encodes a Cas9 protein under the control of a constitutive baculovirus IE1 promoter and gRNA expression cassettes under the control of U6 promoters. The *immediate early-1* (*ie-1*) and *me53* genes of BmNPV were selected as targets, and two gRNAs were designed to target each of them separately. TG silkworms carrying such a construct showed significant improvement in viral resistance with a significant reduction in both viral DNA copy numbers and gene expression after inoculation with BmNPV occlusion bodies (OBs). This report of successful *in vivo* antiviral research using the TG CRISPR/Cas9 system in the context of natural viral infection in animals contributes to modern sericulture and provides the basis for future antiviral therapy in other species.

## RESULTS

### TG CRISPR/Cas9 system construction.

**FIG 1:**
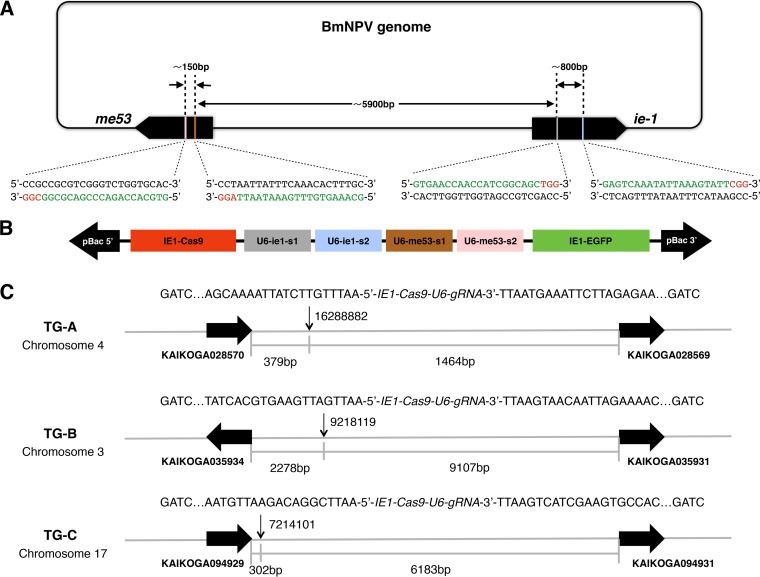
Construction of the BmNPV-specific CRISPR/Cas9 system in the silkworm. (A) Schematic representation of the location of gRNA-targeted sequences. The black rounded square represents the BmNPV genome, and the two large black arrows represent the *me53* and *ie-1* genes. The gRNA target sequences are green, and the PAM sequences are red. The interspace distances between pairs of the four target sites are indicated in base pairs. (B) Design of the pBac-EGFP-Cas9/4×gRNAs construct. The red box represents the Cas9 expression cassette. The gray, blue, brown, and pink boxes represent gRNA expression cassettes targeting ie1-s1, ie1-s2, me53-s1, and me53-s2, respectively. The green box represents the EGFP expression cassette. (C) Genomic insertions of the pBac-EGFP-Cas9/4×gRNAs construct in the TG-A, TG-B, and TG-C lines. The vertical arrows indicate the insertion sites of the TG lines. Chromosome localization and partial genomic DNA sequences between the BfuCI site and the 3′ and 5′ insertion boundaries of the pBac-EGFP-Cas9/4×gRNAs construct are shown. The horizontal black arrows represent contiguous genes of the insertion sites, and the interspace distance between the insertion sites of the construct and its contiguous genes is indicated in base pairs.

We selected the *ie-1* and *me53* genes of BmNPV as targets, with two gRNA target sites for each of them separately. The *ie-1* and *me53* genes are separated by a distance of ∼5 kb in the BmNPV genome (Fig. 1A). The *piggy*Bac-based TG plasmid constructed to target *me53* and *ie-1* contained three different types of expression cassettes: (i) Cas9 expressed under the control of the IE1 promoter, (ii) four gRNAs separately driven by the B. mori U6 promoter, and (iii) IE1-driven enhanced green fluorescent protein (EGFP) as the selecting marker. These expression cassettes were assembled to generate a single pBac-EGFP-Cas9/4×gRNAs construct (Fig. 1B). The generation of TG silkworms has been described previously (23). We obtained three TG lines (TG-A, TG-B, and TG-C), and the transgene locations in the genome were identified by inverse PCR and sequencing (Fig. 1C). Compared to wild-type (WT) and heterozygous TG animals, TG homozygotes showed a 2-day developmental delay during the larval stage, although there were no deleterious effects in viability or fecundity, so we used TG heterozygotes in all of our experiments. To detect the nonspecific gene modification events induced by the BmNPV-targeted CRISPR/Cas9 system, cloning and sequencing of the potential off-target sites were conducted in the exonic regions of the TG-A heterozygote genome and no unintended gene disruptions were found (see Table 2).

### TG silkworms exhibit higher resistance to BmNPV infection.

Positive G_1_ silkworms of the TG-A, TG-B, and TG-C lines were crossed with WT moths to obtain G_2_ heterozygous animals for BmNPV infection investigation. For each TG line, a total of 300 newly exuviated third-instar silkworm larvae were divided into five groups and infected separately *per os* with different dosages of BmNPV OBs (0, 10^4^, 10^5^, 10^6^, and 10^7^ OBs/larva). WT larvae at the same developmental stage were treated in parallel as a control. We monitored the percent mortality of each group from 4 to 10 days postinfection (dpi). None of the individuals in the noninfected control groups (0 OBs/larva) died in any of our experiments.

**FIG 2:**
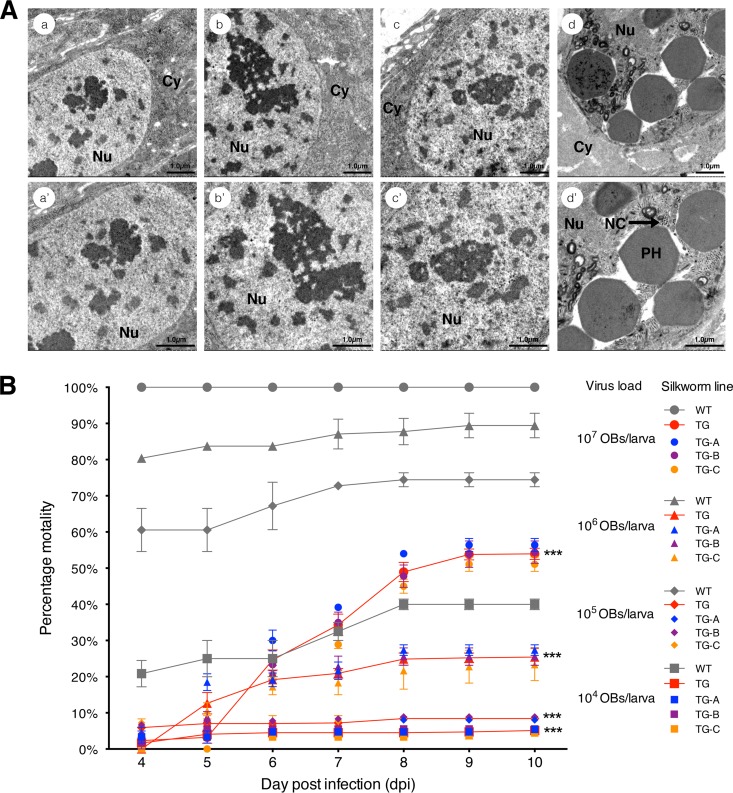
TG silkworms exhibit improved resistance to BmNPV infection. (A) Electron microscopic observations of larval midgut column epithelial cells of TG and WT larvae treated with 10^6^ OBs/larva at 60 hpi. (a to c′) BmNPV-infected TG-A, TG-B, and TG-C larval midgut column epithelial cells at 60 hpi and enlarged image. (d and d′) BmNPV-infected WT larval midgut column epithelial cells at 60 hpi and enlarged image. The arrow points to a cross-section of a nucleocapsid bundle in the nucleus. Nu, nucleus; Cy, cytoplasm; PH, polyhedron; NC, nucleocapsid. Scale bars, 1.0 μm. (B) Mortality percentages after oral inoculation with different doses of BmNPV OBs. Red lines with filled circles, triangles, diamonds, and squares indicate the average mortality percentages of TG-A, TG-B, and TG-C silkworms after oral inoculation with BmNPV at 10^7^, 10^6^, 10^5^, and 10^4^ OBs/larva, respectively. The respective mortality percentages of TG-A, TG-B, and TG-C silkworms inoculated with different doses of OBs are indicated separately with corresponding blue, purple, and orange symbols. Gray lines indicate the mortality percdentages of WT silkworms. The mortality percentages of the TG-A, TG-B, TG-C, and WT groups are the averages of triplicate data. The data shown represent the mean ± SEM. ***, *P* < 0.001 by two-tailed Student *t* test.

At 60 h postinfection (hpi), 82% (*n* = 60) of the TG-A larvae treated with 10^6^ OBs/larva developed normally without signs of disease. In contrast, 80% (*n* = 60) of the WT animals treated with 10^6^ OBs/larva showed enhanced locomotor activity and took on a yellow, puffy appearance with swelling of the segmental membrane at 60 hpi (24, 25). Ultrathin sections of midgut columnar epithelial cells of TG silkworms treated with 10^6^ OBs/larva and WT silkworms treated with 10^6^ OBs/larva were observed at 60 hpi by transmission electron microscopy. No nucleocapsids or polyhedra were found in the nuclei of the midgut columnar epithelial cells of BmNPV-infected TG-A, TG-B, and TG-C silkworms (Fig. 2A, a to c′). In contrast, rod-shaped nucleocapsids and polyhedra were found to accumulate in the nuclei of the midgut columnar epithelial cells of BmNPV-infected WT silkworms (Fig. 2A, d and d′).

The percent mortality of WT larvae at 10 dpi was significantly higher than that of TG larvae after inoculation with different doses of OBs (*P* = 1.952e-05, 4.643e-06, 5.714e-05, and 7.478e-06 for treatment with 10^4^, 10^5^, 10^6^, and 10^7^ OBs/larva, respectively [two-tailed Student *t* test]). The percent mortality increased to 40, 74, 89, and 100% (*n* = 60 for each) at 10 dpi in a dose-dependent manner in WT silkworms infected with BmNPV at 10^4^, 10^5^, 10^6^, and 10^7^ OBs/larva, respectively; In contrast, the average mortality percentages of the three TG lines (TG-A, TG-B, and TG-C) were only 5, 8, 25, and 54% (*n* = 60 for each), respectively (*P* < 0.001 by two-way analysis of variance [ANOVA]) (Fig. 2B). Interestingly, TG silkworms infected with the largest viral dose (10^7^ OBs/larva) had a lower feeding rate than TG silkworms treated with 10^6^ OBs/larva and thus displayed a delayed onset of BmNPV disease. These results demonstrate that TG animals showed significantly higher resistance to BmNPV infection.

### BmNPV-specific CRISPR/Cas9 system induces targeted genomic mutagenesis.

**FIG 3:**
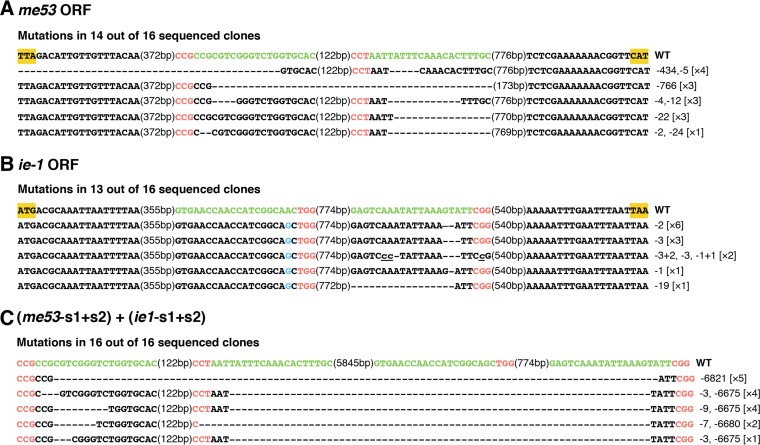
Mutations recovered from CRISPR/Cas9-mediated disruption of BmNPV target sites. Total DNA extracted from six TG and six WT larvae treated with 10^6^ OBs/larva at 48 hpi was used as the template to amplify viral DNA sequences spanning the target sites. The resultant amplified fragments were cloned and sequenced. Mutations in *me53* (A), *ie-1* (B), and interspace fragments between me53-s1 and ie1-s2 (C) are shown. For each gene, the open reading frame (ORF) in the WT sequence is shown at the top with the start and stop codons highlighted in yellow, the target sites in green, and the PAM sequences in red. The blue letters indicate the SNP in the ie1-s1 target sequence. The value in parentheses between every two sequences refers to the length of the interspace fragment in base pairs. Deletions are shown as dashes, and insertions are shown as lowercase letters highlighted as underlined text. The net change in length caused by each indel mutation is enumerated to the right of each sequence (+, insertion; −, deletion). The number of times each mutant clone was isolated is shown in brackets.

CRISPR/Cas9-induced double-strand breaks (DSBs) can be repaired by the error-prone nonhomologous end joining (NHEJ) pathway, thus creating mutations at the genomic level (26, 27). To verify that the significantly decreased mortality rate of BmNPV-infected TG silkworms resulted from the CRISPR/Cas9-mediated cleavage of viral genomes, we performed PCR-based analyses to investigate the genomic mutations in the targeted loci of the BmNPV genome. The total DNA samples extracted from six treated (10^6^ OBs/larva) TG-A larvae and six treated (10^6^ OBs/larva) WT larvae at 48 hpi were subjected to gene amplification separately. Fragments spanning four targeting sites (ie1-s1, ie1-s2, me53-s1, and me53-s2) were amplified and sequenced. The mutations detected included small and large deletions (Fig. 3). In addition, the ie1-s1 target site did not exhibit any mutation patterns. Analysis of the target sequence of ie1-s1 revealed that a single nucleotide polymorphism (SNP) occurred in the BmNPV genome used in our biological assay, although the other 22 bases in the 23-bp target sequence matched perfectly. The remaining three gRNA target sequences (ie1-s2, me53-s1, and me53-s2) are conserved in all BmNPV genotypes and manifested a high mutagenesis frequency. Most of the nucleotide deletion events (introduced by a single gRNA) and small-segment deletion events (introduced by two gRNAs with nearby target sites) detected in a single gene caused a frameshift and abrogated the function of the *ie-1* and *me53* genes (Fig. 3A and B). Moreover, PCR amplification and subsequent Sanger sequencing of the whole region spanning the *me53* and *ie-1* genes (with primers me53-F and ie1-R) showed that the deletion of large segments (∼7 kb) between two long-distance target sites (me53-s1 and ie1-s2; me53-s2 and ie1-s2) resulted in the knockout of six genes between the *ie-1* and *me53* genes, including *ie0*, *odv-e18*, *odv-ec27*, and three unidentified genes in the BmNPV genome (Fig. 3C). We anticipate that these mutant BmNPV genomes resulting from NHEJ repair might compromise viral DNA replication, nucleocapsid formation, and the consequent efficient production of budded virus (BV) and occlusion-derived virus.

### The CRISPR/Cas9 system facilitates BmNPV clearance.

**FIG 4:**
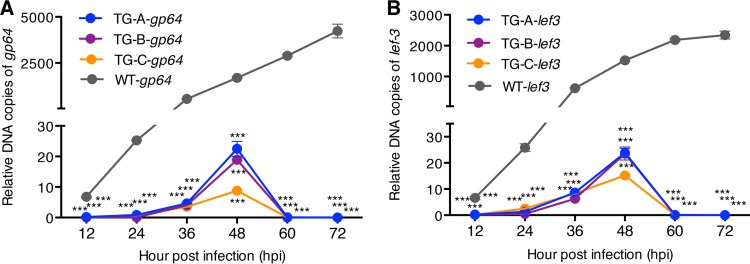
Relative numbers of copies of BmNPV genes after viral inoculation with 10^6^ OBs/larva. The total DNA extracted every 12 h (0 to ∼72 hpi) from TG-A, TG-B, TG-C, and WT larvae treated with 10^6^ OBs/larva was used as a template with *gp64* and *lef-3* as the detection indicators for qPCR analysis of the accumulated viral DNA levels. Blue, purple, orange, and gray lines with filled circles indicate relative numbers of copies of *gp64* and *lef-3* DNA in TG-A, TG-B, TG-C, and WT silkworms, respectively. Data represent the mean ± SEM. ***, *P* < 0.001 by two-tailed Student *t* test.

We investigated the relative number of DNA copies of BmNPV genes to monitor viral proliferation in TG and WT silkworms. Total DNA was extracted from silkworms treated with 10^6^ OBs/larva in the TG-A, TG-B, TG-C, and WT groups every 12 h (from 0 to 72 hpi). Several BmNPV genes, including *lef-1*, *lef-3*, *gp41*, and *gp64*, were used in previous studies for viral DNA quantification (8, 9, 28). Among them, the *lef-3* and *gp64* genes, two essential genes beyond the range of the gRNA target deletion sequences, were selected as the detection indicators in quantitative PCR (qPCR) analyses of the relative abundance of BmNPV with *Bmrp49* as the internal reference gene. The relative numbers of copies of *gp64* and *lef-3* in WT silkworms were significantly higher (*P* < 0.001, two-tailed Student *t* test) than those of TG-A, TG-B, and TG-C silkworms at all times after BmNPV infection (from 12 to 72 hpi) (Fig. 4). The relative numbers of copies of these two indicator genes were apparently increased in both the TG and WT groups at 48 hpi, which corresponded to the time when the relative abundance of BmNPV in the TG group reached its highest level. At 60 hpi, the relative numbers of copies of BmNPV genes continued to increase in the WT group, while in contrast, abundance was reduced to low levels in the TG groups. The relative copy numbers of the *gp64* and *lef-3* genes at 72 hpi in WT silkworms were more than 10^5^- and 10^4^-fold higher, respectively, than those in TG silkworms. These data showed a robust reduction in the relative abundance of BmNPV in TG silkworms, indicating that CRISPR/Cas9-mediated disruption facilitated the clearance of BmNPV in the latter.

### The expression levels of different-phase BmNPV genes significantly decrease.

**FIG 5:**
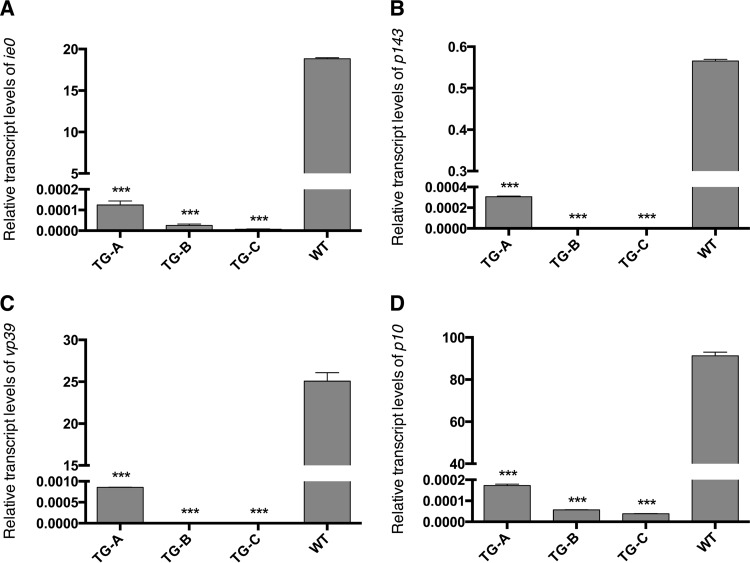
Expression levels of different-phase BmNPV genes in TG and WT silkworms treated with 10^6^ OBs/larva at 60 hpi. The relative transcript levels of *ie0* (A), *p143* (B), *vp39* (C), and *p10* (D) are shown. Data represent the mean ± SEM. ***, *P* < 0.001 by two-tailed Student *t* test.

To confirm that the BmNPV-specific CRISPR/Cas9 system could effectively inhibit BmNPV gene expression, we investigated the transcription levels of four BmNPV genes, namely, an immediate early gene (*ie0*), a delayed early gene (*p143*), a late gene (*vp39*), and a hyperexpressed late gene (*p10*), which correspond to four different phases in the BmNPV gene temporal expression pattern. Total RNA extracted from larvae in the TG-A, TG-B, TG-C, and WT groups treated with 10^6^ OBs/larva at 60 hpi was used for real-time qPCR analysis. Expression of the four genes in TG-A, TG-B, and TG-C animals decreased to a nearly undetectable level compared to that in WT animals (Fig. 5), indicating that the CRISPR/Cas9 system disrupted BmNPV genome proliferation and subsequent mRNA expression.

## DISCUSSION

The CRISPR/Cas9 system is derived from bacterial defense systems and functions to combat viruses (29–31). In a wide variety of bacteria and archaea, CRISPR loci have been found to confer acquired resistance to invading viruses, as well as other foreign plasmids, by targeting nucleic acids in a sequence-specific manner. The CRISPR/Cas9 system was used to disrupt the viral genomes of HIV-1 and HBV in human cell lines and mouse models (11, 12, 15, 16). However, there are limitations in the evaluation of the feasibility and long-term impact of CRISPR/Cas9 systems in antiviral therapy when using cell culture and animal models of human disease, as these are not likely to mimic all of the aspects of a natural viral infection in a real biological host, and a transient defense system may not sustain resistance. Here we established an inheritable BmNPV-specific CRISPR/Cas9 system in silkworms that has proved to offer permanent protection in a real host-virus battle.

Previous TG RNAi-based antiviral strategies were designed to act posttranscriptionally to destroy specific viral mRNAs, and these inhibit BmNPV replication to some extent but fail to target or eliminate the viral genome DNA pool (5, 6). However, the CRISPR/Cas9 system provides an RNA-guided editing tool for site-specific DNA cleavage of viral genomes. Small-deletion mutagenesis induced by a single gRNA may lead to frameshift mutations and disable essential viral genes. The CRISPR/Cas9 machinery can also achieve large-segment deletions by using two long-distance target sites and result in the simultaneous knockout of several genes and cause severe damage to the virus replicative templates. Moreover, we suspect that large-scale viral genome disruptions introduced by multiple gRNA targeting may systematically destroy viral genomes (particularly small viral DNA genomes) without possible reactivation. Although DSB formation activates DNA repair mechanisms, we presume that the damage is too extensive to be repaired and the fragmented segments that result from multiplex cleavage may potentially undergo degradation.

We designed only four BmNPV target sites, which we expected to offer an optimal compromise between cleavage frequency and concomitant off-target risks. Two target sites were specific to the early gene *ie-1*, a core baculovirus DNA replication gene, to effectively inactivate the viruses, and the other two target sites were specific to the *me53* gene, another early-transcribed gene that is located ∼5 kb away from *ie-1*, to produce large-segment deletions. Although one target site for the *ie-1* gene abolished genomic cleavage because of a single-nucleotide mismatch between the gRNA template and its BmNPV genome target, our data showed that the remaining three target sites were sufficient to introduce large-segment deletions into BmNPV genomes and the relative abundance of BmNPV was decreased to a nearly undetectable level (>10^4^-fold) in TG silkworms (Fig. 4), which was quite different from the limited viral blockage (2.5- to ∼16.7-fold) achieved by RNAi-based antiviral strategies (8, 9). The markedly enhanced survival of the engineered silkworms supports the BmNPV-specific CRISPR/Cas9 system as a promising tool for application in sericulture. However, TG homozygotes showed a developmental delay that was probably due to the toxic effect induced by high Cas9 and gRNA expression levels (32–34). Such unexpected side effects should be comprehensively considered in future applications of any CRISPR/Cas9-based antiviral strategy.

In summary, we demonstrated that a BmNPV-specific CRISPR/Cas9 system integrated into the silkworm genome conferred a heritable immune defense against BmNPV invasion of B. mori. We provide evidence of CRISPR/Cas9-mediated *in vivo* pathogen DNA disruption without the use of replication-competent plasmids or cell lines. The significantly enhanced BmNPV resistance in genetically engineered silkworms revealed this TG CRISPR/Cas9 system as a promising antiviral strategy that should shed light on future antiviral research.

## MATERIALS AND METHODS

### Silkworm strain and virus stock.

The multivoltine, nondiapausing Nistari strain was used in all of our experiments. Larvae were reared on fresh mulberry leaves under standard conditions. The WT Zhejiang strain of BmNPV (GenBank accession no. JQ991008) was propagated in fifth-instar larvae and used for infection. The OBs were harvested from the infected hemolymph before the larvae died. The virus stock was prepared as described previously (35). The OBs were counted with a Neubauer hemocytometer. The virus stock, at a concentration of 10^9^ OBs/ml, was diluted serially to prepare inocula with concentrations of 10^8^, 10^7^, and 10^6^ OBs/ml.

### Target gene selection and vector construction.

The *ie-1* gene is essential for Autographa californica multiple nucleopolyhedrovirus (AcMNPV) DNA replication (36), and the *me53* gene is required for efficient BV production (37, 38). Both genes were reported to be transcriptionally active as early as 1 hpi in AcMNPV (39). We selected these two genes in BmNPV as the targets since they are major early-transcribed genes that are involved with baculovirus propagation, and they are separated by a distance of ∼5 kb in the BmNPV genome, which tended to produce large-fragment deletions by the CRISPR/Cas9 machinery (40). We searched the *ie-1* and *me53* gene sequences in the BmNPV genome for potential 20-base targeting sequences and chose four candidates that are compatible with consensus gRNA target sites (GN19NGG): ie1-s1 (5′-GTGAACCAACCATCGGCAACTGG-3′) and ie1-s2 (5′-GAGTCAAATATTAAAGTATTCGG-3′) for the *ie-1* gene and me53-s1 (5′-CCGCCGCGTCGGGTCTGGTGCAC-3′) and me53-s2 (5′-CCTAATTATTTCAAACACTTTGC-3′) for the *me53* gene. To avoid unintended gene disruptions in silkworms, we performed blast searches for the four targeting sequences in the silkworm genome in KAIKObase (http://sgp.dna.affrc.go.jp/KAIKObase/). The potential genomic off-target loci that were most similar to the gRNA targeting sequences had more than three mismatches, indicating that these targeting sequences can be used for BmNPV targeting in silkworms (32). A constitutive Cas9 expression cassette under the control of a baculovirus immediate early IE1 gene promoter and four targeting cassettes expressing the gRNAs targeting *ie-1* and *me53* under the separate control of the silkworm small nuclear RNA promoter (U6 promoter, a ubiquitous polymerase III promoter derived from the upstream sequence of the B. mori U6-2 small nuclear RNA gene) were constructed through a series of cloning steps to generate pBac-EGFP-Cas9/4×gRNAs. Cloning of the IE1-Cas9 and U6 promoters was carried out as described previously (41–43). All of the primers used for vector construction are listed in Table 1.

**TABLE 1:** Primers used in this study

Expt and primer name	Primer sequence (5′–3′)
Plasmid construction	
IE1-Cas9_F	GACAAGAGACGTCGCTAGCGGCCACCATGGACAAGAAGTACTCCAT
IE1-Cas9_R	CTGATTATGATCTAGAGTCGTCACACCTTCCTCTTCTTCTTGGGGT
U6-2_F	AGGTTATGTAGTACACATTG
U6-2_R	ACTTGTAGAGCACGATATTTTG
ie1-sg1-R	GCTATTTCTAGCTCTAAAACGTTGCCGATGGTTGGTTCACACTTGTAGAGCACGATATTTTG
ie1-sg2-R	GCTATTTCTAGCTCTAAAACAATACTTTAATATTTGACTCACTTGTAGAGCACGATATTTTG
me53-sg1-R	GCTATTTCTAGCTCTAAAACAATTATTTCAAACACTTTGCACTTGTAGAGCACGATATTTTG
me53-sg2-R	GCTATTTCTAGCTCTAAAACCCGCGTCGGGTCTGGTGCACACTTGTAGAGCACGATATTTTG
U6-sgRNA-R	AAAAAAGCACCGACTCGGTGCCACTTTTTCAAGTTGATAACGGACTAGCCTTATTTTAACTTGCTATTTCTAGCTCTAAAAC
Dimer1_Bsa1-F	ATGCGGTCTCCTGACCCTAGGAGGTTATGTAGTACACATTG
Dimer1_Bsa1-R	ATGCGGTCTCGTAGGAAAAAAGCACCGACTCGGTGCCACT
Dimer2_Bsa1-F	ATGCGGTCTCACCTAAGGTTATGTAGTACACATTG
Dimer2_Bsa1-R	ATGCGGTCTCGCAAGAAAAAAGCACCGACTCGGTGCCACT
Dimer3_Bsa1-F	ATGCGGTCTCACTTGAGGTTATGTAGTACACATTG
Dimer3_Bsa1-R	ATGCGGTCTCGTAATAAAAAAGCACCGACTCGGTGCCACT
Dimer4_Bsa1-F	ATGCGGTCTCAATTAAGGTTATGTAGTACACATTG
Dimer4_Bsa1-R	ATGCGGTCTCGGAGTCCTAGGAAAAAAGCACCGACTCGGTGCCACT
Relative BmNPV gene copy no. detection	
gp64-qPCR-F	CTTTAATGAGCAGACACGCAG
gp64-qPCR-R	CTTTAATGAGCAGACACGCAG
lef3-qPCR-F	CGGAAGAGGTAGAACGGTCA
lef3-qPCR-R	GTGCGAGGCTAAAGAAAACG
BmRP49-qPCR-F	AAACATACAAGATGGCTATAAGACCTG
BmRP49-qPCR-R	TTTATAAATGACATGTGAACATACCTC
Relative BmNPV gene expression level detection	
ie1-qRT-F	GCTCAAGACCACTGATAATCTC
ie1-qRT-R	AATCGTCCAAGTATTCGTCCA
me53-qRT-F	TCTTTAACTTCCGTCGTGTG
me53-qRT-R	ACGATTCTGACCGCATAGAC
ie0-qRT-F	ATGATAAGAACCAGCAGTCAC
ie0-qRT-R	CTTTGCGTCGATTTGTATGTC
p143-qRT-F	TGGCTTCATACTTTAGCAACC
p143-qRT-R	GTTTGACGATGACAACCACAG
vp39-qRT-F	TCTAAATCTCAATTCCTCCGTG
vp39-qRT-R	GCATTCTAGACACCACAAACC
p10-qRT-F	CCATTGCGGAAACTAACACA
p10-qRT-R	AGCAGTGTCACCGGTCAATA
Bmrp49-qRT-F	TCAATCGGATCGCTATGACA
Bmrp49-qRT-R	ATGACGGGTCTTCTTGTTGG
Mutagenesis detection	
ie1-F	GTGTTCGCCATTAGGGCAGTA
ie1-R	GCGCACCAACTCCCATTGTT
me53-F	GGTCTACCTCTAACAGCGTCG
me53-R	TAAAGCCTCTCGATGGCTGA
Inverse PCR	
Inverse PCR-F1	CAGTGACACTTACCGCATTGA
Inverse PCR-R1	CATTTTGACTCACGCGGTC
Inverse PCR-F2	CGCTATTTAGAAAGAGAGAGCAA
Inverse PCR-R2	ATCACGTAAGTAGAACATGAAATAACA
Off-target assay	
ie1-s1-OT1-F	CTTGGACCAATTAGGACGTGG
ie1-s1-OT1-R	GAGTCTCGTGCGCCATTTGT
ie1-s1-OT2-F	ACTGTACATCATGCACGGGG
ie1-s1-OT2-R	CCCACCCCAAATCAATACGC
ie1-s1-OT3-F	ATTCAGTGCGTCACGTAGGG
ie1-s1-OT3-R	GAATATGCAGTGCGAAGGCG
ie1-s2-OT1-F	CAGACCGCAAAGCTCACAGA
ie1-s2-OT1-R	TTTGCTACCTAACGCCTCCA
ie1-s2-OT2-F	ACGCCTCTAAGTCCAAGTCC
ie1-s2-OT2-R	GACTTCGGTAGGGCAAGGAT
ie1-s2-OT3-F	GGGTCGTATAGCCGAACTGG
ie1-s2-OT3-R	GCGACTCGAAAACAGCTTCC
me53-s1-OT1-F	AAGGAACTGTCGGCGAAAAGA
me53-s1-OT1-R	GCGAGCGTCTTGTGTGAGAG
me53-s1-OT2-F	GACTGTCGCTAGTGAGGAGC
me53-s1-OT2-R	TCGCCCGCACATACATACAA
me53-s1-OT3-F	TGATGCGTTAAAAGCGCGAG
me53-s1-OT3-R	GTGGATAGTCGGCCAGATCG
me53-s2-OT1-F	CGAGAAGTCAAGCTGAGCGA
me53-s2-OT1-R	ATGTCCGGAGTCCAGTAGCA
me53-s2-OT2-F	TGCCCGCAGCTTGTAAGTAT
me53-s2-OT2-R	GTTGGAAGGGGCAGACTTGA
me53-s2-OT3-F	TTCTTTCTCCGTGCACACCA
me53-s2-OT3-R	GTGTTCGTTTGCCTGTCGAG

### Silkworm genetic transformation.

DNA solutions containing pBac-EGFP-Cas9/4×gRNAs and the helper plasmids (44) were microinjected into preblastoderm G_0_ embryos that were then incubated at 25°C in a humidified chamber for 10 to 12 days until larval hatching. Larvae were reared on fresh mulberry leaves under standard conditions. Putative TG G_0_ adults were mated with WT moths to produce G_1_ progenies, and G_1_ progenies were scored for the presence of the EGFP-encoding marker gene by fluorescence microscopy (Nikon AZ100). Inverse PCR was carried out as described previously to investigate the genomic insertion loci of the transgene (23), and the primers used for detection are listed in Table 1.

### Off-target effect assays.

To evaluate the off-target effects in TG silkworms, we selected three exonic off-target sequences with perfect matches for the seed sequence (8 to 13 nucleotides) adjacent to the protospacer adjacent motif (PAM) with CRSPRdirect (http://crispr.dbcls.jp/) (45) and KAIKObase for each of the four target sites. TG silkworm genomic DNA was used as a substrate to amplify putative off-target regions, and amplicons were cloned, sequenced, and compared to the WT silkworm genome. The off-target candidates are listed in Table 2, and the sequences of the primers used for detection are listed in Table 1.

**TABLE 2:** Off-target analysis of BmNPV-targeted CRISPR/Cas9 system on TG silkworm endogenous genes^*a*^

Target site	Sequence	Partial complementarity	Load ID	Position	Gene description	Off-target ratio
ie1-s1	GTGAACCAACCATCGGCAGCTGG					
OT1	CAGTGCCGGCCC**TCGGCAGC**GGG	8+PAM	BMgn000871	chr13: 5171945..5186942 (+ strand)	Putative uncharacterized protein GLEAN_07583	0/24
OT2	GTGGTGACACCG**TCGGCAGC**TGG	8+PAM	BMgn002354	chr21: 5590712..5595774 (− strand)	Endoprotease FURIN	0/18
OT3	GCACATCCCGCG**TCGGCAGC**CGG	8+PAM	BMgn001361	chr2: 9790024..9793483 (− strand)	Putative uncharacterized protein (fragment)	0/12
ie1-s2	GAGTCAAATATTAAAGTATTCGG					
OT1	GTATAAC**ATATTAAAGTATT**TGG	13+PAM	BMgn016383	chr17: 4311187..4315556 (− strand)	Ribosomal protein L29	0/15
OT2	TGGGGGCT**TATTAAAGTATT**AGG	12+PAM	BMgn017252	chr26: 11357548..11458566 (+ strand)	Antibacterial peptide	0/24
OT3	TAATTTACAACA**AAAGTATT**GGG	8+PAM	BMgn000864	chr13: 4620587..4632622 (+ strand)	Viral A-type inclusion protein	0/22
me53-s1	CCGCCGCGTCGGGTCTGGTGCAC					
OT1	CCT**CCGCGTCGG**CAAAAACTATC	9+PAM	BMgn001199	chr13: 9102828..9103217 (− strand)	Expressed protein (fragment)	0/22
OT2	CCC**CCGCGTCG**CGTCGCACGGAG	8+PAM	BMgn006670	chr10: 88015..89899 (− strand)	Putative aphid transmission factor	0/19
OT3	CCT**CCGCGTCG**TCGTGAAGATCG	8+PAM	BMgn006704	chr10: 2770894..2772063 (+ strand)	Plasminogen activator inhibitor 1 RNA-binding protein	0/23
me53-s2	CCTAATTATTTCAAACACTTTGC					
OT1	CCA**AATTATTTCAAA**GCCGTTTG	12+PAM	BMgn008313	chr18: 11728909..11762529 (− strand)	Transcriptional regulator ATRX (X-linked helicase II)	0/24
OT2	CCA**AATTATTTCAAA**TCTTCAAT	12+PAM	BMgn017245	chr26: 8903443..8905154 (+ strand)	Putative uncharacterized protein (fragment)	0/24
OT3	CCC**AATTATTTCA**CAGCTCCCAG	10+PAM	BMgn006654	chr10: 1712172..1740556 (− strand)	Putative uncharacterized protein	0/17

a OT indicates off-target sites. Perfect matches in the seed sequence adjacent to the PAM are in bold, and the number of matched bases is listed. Genes in which OT were located are shown, together with the load ID and position on the chromosome. All of the OT sequences listed were confirmed by cloning and sequencing with WT Nistari silkworms, and intronic OT and candidates with mutations between WT Nistari genome and the sequence in KAIKObase have been removed.

### Viral inoculation and mortality analyses.

Newly exuviated third-instar larvae were starved for 12 h before viral inoculation. Each larva was fed a 1-cm^2^ piece of mulberry leaf that had been smeared with 10 μl of an OB suspension. Only those larvae that consumed the entire leaf piece after 24 h of feeding were maintained. Virus-fed larvae were subsequently reared on fresh mulberry leaves and observed for signs of infection. Dead larvae were removed immediately to prevent horizontal contamination. Mortality was recorded daily until 10 dpi. The TG lines were mated with the WT to generate heterozygous offspring for viral inoculation. The resistance of each TG line was investigated via oral infection with wild BmNPV at four different polyhedron doses, 10^7^, 10^6^, 10^5^, and 10^4^ OBs/larva, achieved by applying 10 μl of OB suspensions with concentrations of 10^9^, 10^8^, 10^7^, and 10^6^ OBs/ml to a fresh mulberry leaf. In each infected group of a TG line, 60 newly exuviated third-instar TG larvae were each provided a leaf and monitored to ensure that they consumed it completely. The noninfected group (0 OBs/larva), also including 60 larvae, was fed on leaves treated with an equal volume of sterile water. WT larvae were treated in parallel as a control.

### Transmission electron microscopy.

Midguts dissected from TG-A, TG-B, TG-C, and WT silkworms treated with 10^6^ OBs/larva at 60 hpi were cut into 1- to 2-mm fragments that were then fixed with 2.5% glutaraldehyde in 0.1 M phosphate buffer (pH 7.2) overnight at 4°C; washed with phosphate buffer, postfixed with 1% osmium tetroxide in phosphate buffer for 1 to 2 h, washed again, dehydrated with a graded series of ethanol concentrations, and then embedded in Embed812 resin. Ultrathin sections (60 to 90 nm) were stained in 2% uranyl acetate (pH 5.0), followed by 10 mM lead citrate (pH 12), and viewed with a Hitachi H-7650 transmission electron microscope. The midguts from more than six larvae in each group were cut up.

### qPCR analysis of real-time virus accumulation levels after BmNPV oral infection.

The real-time virus accumulation level was measured by viral DNA quantification. Whole larval samples were separately collected from silkworms in the TG-A, TG-B, TG-C, and WT groups treated with 10^6^ OBs/larva (six larvae as a sample in each group) once every 12 h (from 0 to 72 hpi) and ground with a tissue lyser before DNA extraction. Total DNA was extracted with a Tissue DNA kit (OMEGA) and then treated with RNase A (Thermo). The DNA templates (60 ng) were amplified with primers for the BmNPV *gp64* and *lef-3* genes. The B. mori ribosomal protein 49 gene (*Bmrp49*) was used as an internal control to standardize the variance of the different templates. qPCR analysis was performed with an Eppendorf Mastercycler ep realplex with Toyobo SYBR green Real-Time PCR master mix. The amplification program used was incubation at 95°C for 3 min, followed by 40 cycles of 95°C for 15 s and 60°C for 1 min. Standard curves were determined with 10-fold serially diluted DNA. The data were analyzed by GraphPad Prism version 6. The sequences of all of the qPCR primers used are listed in Table 1. The test was performed three times.

### Detection of the expression levels of different-phase BmNPV genes.

Whole larval samples were separately collected from silkworms treated with 10^6^ OBs/larva in the TG-A, TG-B, TG-C, and WT groups (six larvae as a sample in each group) at 60 hpi and ground with a tissue lyser before RNA extraction. Total RNA was extracted with TRIzol reagent (Invitrogen) and then treated with RNase-free DNase I (TaKaRa). One microgram of total RNA was used as the template to synthesize cDNA with the HiScript RT-Q SuperMix for qPCR (+gDNA wiper) (Vazyme). Quantitative mRNA measurements were performed with the Toyobo SYBR green Real-Time PCR master mix in an Eppendorf Mastercycler ep realplex. The PCR program was incubation at 95°C for 3 min, followed by 40 cycles of 95°C for 15 s and 60°C for 1 min. Standard curves were determined with 10-fold serially diluted cDNA. The test was performed in triplicate. The data were normalized to *Bmrp49* and analyzed by GraphPad Prism version 6. The sequences of all of the reverse transcription-qPCR primers used are included in Table 1.

### Mutagenesis analysis.

Total DNA samples extracted from six whole larvae treated with 10^6^ OBs/larva in the TG-A group and six larvae treated with 10^6^ OBs/larva in the WT group were subjected to amplification with BmNPV-specific primers at 48 hpi, followed by cloning and sequencing of the resultant BmNPV DNA fragments. The following primers were designed to detect mutagenesis at targeted sites. me53-F and me53-R detected mutagenesis at targeting sites 1 and 2 in the *me53* gene, ie1-F and ie1-R detected mutagenesis at targeting sites 1 and 2 in the *ie-1* gene, and me53-F and ie1-R detected mutagenesis spanning the *me53* and *ie-1* genes with four targeting sites. The amplification program was incubation at 95°C for 5 min, followed by 35 cycles of 94°C for 30 s, 55°C for 30 s, and 72°C for 8.5 min and ending with 72°C for 10 min. The sequences of the primers used are listed in Table 1.

### Statistical analysis.

All of the experiments in this study were performed with at least three replicates. All data are expressed as the mean ± the standard error of the mean (SEM). The differences between groups were examined by either two-tailed Student *t* test or two-way ANOVA. All statistical calculations and graphs were made with GraphPad Prism version 6. Statistically significant differences are indicated by asterisks.
